# Supplementary material for: Single Cell Transcriptomic and Chromatin Profiles Suggest Layer Vb Is the Only Layer With Shared Excitatory Cell Types in the Medial and Lateral Entorhinal Cortex
Source: Front Neural Circuits. 2022 Jan 26;15:806154. doi: 10.3389/fncir.2021.806154 (PMC8826650; doi:10.3389/fncir.2021.806154)
Supplement: Supplementary file 14 [file Data_Sheet_1.ZIP › Inh_vs_Exc_Motifs/homerResults.html]

Inh\_vs\_Exc\_Motifs// - Homer de novo Motif Results


# Homer *de novo* Motif Results (Inh\_vs\_Exc\_Motifs//)

Known Motif Enrichment Results  
Gene Ontology Enrichment Results  
If Homer is having trouble matching a motif to a known motif, try copy/pasting the matrix file into
STAMP  
More information on motif finding results: HOMER
| Description of Results
| Tips
  
Total target sequences = 12244  
Total background sequences = 38563  
\* - possible false positive  

|  |  |  |  |  |  |  |  |  |
| --- | --- | --- | --- | --- | --- | --- | --- | --- |
| Rank | Motif | P-value | log P-pvalue | % of Targets | % of Background | STD(Bg STD) | Best Match/Details | Motif File |
| 1 | G A C T C T A G A G T C C G T A A C T G A G T C A C G T A C T G A T C G T G C A | 1e-194 | -4.470e+02 | 33.83% | 22.09% | 236.7bp (323.3bp) | BHLHE22(var.2)/MA1635.1/Jaspar(0.942) More Information \| Similar Motifs Found | motif file (matrix) |
| 2 | C A T G A T C G G A C T C T G A C G T A A G C T A G C T C T G A | 1e-141 | -3.265e+02 | 35.51% | 25.16% | 233.5bp (294.9bp) | MEOX2/MA0706.1/Jaspar(0.948) More Information \| Similar Motifs Found | motif file (matrix) |
| 3 | C G T A A T C G A C G T A C T G G T C A A G T C G T A C A G C T G A C T T C A G | 1e-85 | -1.980e+02 | 17.86% | 11.75% | 269.2bp (353.6bp) | THRb(NR)/Liver-NR1A2-ChIP-Seq(GSE52613)/Homer(0.944) More Information \| Similar Motifs Found | motif file (matrix) |
| 4 | C A T G G T A C G A C T C G T A C G A T C G A T G A C T G C A T G A C T C T G A A C T G G T A C | 1e-81 | -1.875e+02 | 14.94% | 9.48% | 205.5bp (250.8bp) | Mef2d(MADS)/Retina-Mef2d-ChIP-Seq(GSE61391)/Homer(0.984) More Information \| Similar Motifs Found | motif file (matrix) |
| 5 | G A C T A C T G G T A C C G A T A C T G C G T A A C G T A G C T A T G C C T A G | 1e-58 | -1.355e+02 | 47.67% | 40.41% | 242.3bp (317.0bp) | MafA(bZIP)/Islet-MafA-ChIP-Seq(GSE30298)/Homer(0.893) More Information \| Similar Motifs Found | motif file (matrix) |
| 6 | C T G A C T A G T C A G A C T G G A T C G T A C G T C A A G C T A C G T C A T G | 1e-29 | -6.723e+01 | 47.39% | 42.31% | 257.9bp (335.5bp) | NR1H4/MA1110.1/Jaspar(0.777) More Information \| Similar Motifs Found | motif file (matrix) |
| 7 | C T A G T A G C G A T C A T G C G C T A A G C T A C G T A G C T G C T A C A T G | 1e-26 | -6.134e+01 | 20.00% | 16.30% | 253.2bp (325.8bp) | BARHL2/MA0635.1/Jaspar(0.677) More Information \| Similar Motifs Found | motif file (matrix) |
| 8 | G A T C C T A G T A C G T G A C G C A T C A T G T G A C G T C A C T A G A T C G T A G C G C A T | 1e-25 | -5.796e+01 | 7.04% | 4.88% | 267.9bp (405.1bp) | Zic1::Zic2/MA1628.1/Jaspar(0.627) More Information \| Similar Motifs Found | motif file (matrix) |
| 9 | A T C G A T G C C T G A T A C G C T G A A C T G T A C G C T A G A T C G C G T A | 1e-21 | -4.837e+01 | 5.72% | 3.94% | 259.6bp (323.7bp) | PB0128.1\_Gcm1\_2/Jaspar(0.681) More Information \| Similar Motifs Found | motif file (matrix) |
| 10 | G C T A C G A T G C T A T G C A C T G A G T A C G C A T C A T G T G A C G C T A C A T G G A C T | 1e-20 | -4.723e+01 | 11.94% | 9.37% | 230.8bp (318.6bp) | PB0091.1\_Zbtb3\_1/Jaspar(0.666) More Information \| Similar Motifs Found | motif file (matrix) |
| 11 | T G A C G C T A T C A G T A G C A T C G A G T C G C A T A C T G T A G C G C T A T C A G G T C A | 1e-17 | -4.012e+01 | 19.92% | 16.93% | 250.8bp (339.9bp) | PB0091.1\_Zbtb3\_1/Jaspar(0.618) More Information \| Similar Motifs Found | motif file (matrix) |
| 12 | A T C G G T A C T A C G G A T C T A G C G A T C G A C T A G C T A T G C G T C A | 1e-16 | -3.794e+01 | 28.15% | 24.84% | 272.2bp (440.6bp) | ERRg(NR)/Kidney-ESRRG-ChIP-Seq(GSE104905)/Homer(0.692) More Information \| Similar Motifs Found | motif file (matrix) |
| 13 | A T C G C T A G A G T C G A C T G A T C A G T C C T A G A T C G C G T A C T G A | 1e-16 | -3.765e+01 | 1.57% | 0.81% | 286.3bp (304.1bp) | POL013.1\_MED-1/Jaspar(0.693) More Information \| Similar Motifs Found | motif file (matrix) |
| 14 | A G T C G T A C A T C G T A C G A C T G G T A C C G A T C A T G | 1e-16 | -3.757e+01 | 16.89% | 14.21% | 277.7bp (487.3bp) | POL010.1\_DCE\_S\_III/Jaspar(0.704) More Information \| Similar Motifs Found | motif file (matrix) |
| 15 | T G A C C G T A A C T G T G A C G C T A T A C G T C G A C T A G A T G C G A T C G A T C G A C T | 1e-16 | -3.695e+01 | 7.05% | 5.30% | 286.7bp (371.3bp) | ZNF317/MA1593.1/Jaspar(0.688) More Information \| Similar Motifs Found | motif file (matrix) |
| 16 | C A G T T A C G G C T A G C T A C G A T C T A G T A C G T C G A T C G A C G T A C G A T A C G T | 1e-15 | -3.609e+01 | 6.81% | 5.11% | 247.3bp (281.5bp) | NFAT(RHD)/Jurkat-NFATC1-ChIP-Seq(Jolma\_et\_al.)/Homer(0.771) More Information \| Similar Motifs Found | motif file (matrix) |
| 17 | G A T C A C T G A T G C A C G T A C T G A G C T G A C T A G T C T C A G A T C G | 1e-15 | -3.540e+01 | 0.70% | 0.25% | 270.0bp (369.1bp) | ZNF341/MA1655.1/Jaspar(0.769) More Information \| Similar Motifs Found | motif file (matrix) |
| 18 | C T G A T A G C C G A T A C T G C A T G T A C G C G A T T A G C T G C A A G T C C A T G A C T G | 1e-15 | -3.481e+01 | 3.25% | 2.13% | 265.1bp (384.7bp) | RORg(NR)/Liver-Rorc-ChIP-Seq(GSE101115)/Homer(0.722) More Information \| Similar Motifs Found | motif file (matrix) |
| 19 \* | A G C T T A C G C G T A C A G T A C G T T A C G C G T A G A T C | 1e-11 | -2.664e+01 | 22.95% | 20.40% | 247.2bp (311.2bp) | Fos(bZIP)/TSC-Fos-ChIP-Seq(GSE110950)/Homer(0.767) More Information \| Similar Motifs Found | motif file (matrix) |
| 20 \* | T C A G A T G C G T A C T G A C C G A T T A C G A C G T T A C G C T G A G T A C | 1e-10 | -2.511e+01 | 12.31% | 10.42% | 253.1bp (364.6bp) | MITF(bHLH)/MastCells-MITF-ChIP-Seq(GSE48085)/Homer(0.766) More Information \| Similar Motifs Found | motif file (matrix) |
| 21 \* | A G T C A C T G A G T C A G T C A C T G A C T G A C G T A G T C C G T A A G T C A G T C C G T A | 1e-8 | -2.021e+01 | 0.11% | 0.01% | 182.1bp (128.1bp) | Tbx20(T-box)/Heart-Tbx20-ChIP-Seq(GSE29636)/Homer(0.622) More Information \| Similar Motifs Found | motif file (matrix) |
| 22 \* | A G T C G T A C A T G C C T A G A G T C A C T G A C T G A G T C A C T G G A T C | 1e-6 | -1.442e+01 | 0.24% | 0.08% | 325.5bp (601.5bp) | ZIC3/MA0697.1/Jaspar(0.689) More Information \| Similar Motifs Found | motif file (matrix) |
| 23 \* | A C G T A C T G A G T C A C G T A G T C A C T G A C T G C G T A A C T G A C T G | 1e-4 | -1.113e+01 | 0.15% | 0.05% | 296.6bp (125.6bp) | ZBTB14/MA1650.1/Jaspar(0.620) More Information \| Similar Motifs Found | motif file (matrix) |
| 24 \* | A C G T A C G T A C G T A C T G A C T G A G T C A C G T A C G T A C G T A C T G A C T G A G T C | 1e-3 | -7.956e+00 | 0.06% | 0.01% | 222.9bp (153.1bp) | NF1:FOXA1(CTF,Forkhead)/LNCAP-FOXA1-ChIP-Seq(GSE27824)/Homer(0.667) More Information \| Similar Motifs Found | motif file (matrix) |
